# Runx3 regulates iron metabolism via modulation of BMP signalling

**DOI:** 10.1111/cpr.13138

**Published:** 2021-10-06

**Authors:** Hyun‐Yi Kim, Jong‐Min Lee, You‐Soub Lee, Shujin Li, Seung‐Jun Lee, Suk‐Chul Bae, Han‐Sung Jung

**Affiliations:** ^1^ Division in Anatomy and Developmental Biology Department of Oral Biology Oral Science Research Center, BK21 FOUR, Yonsei University College of Dentistry Seoul Korea; ^2^ Department of Biochemistry School of Medicine and Institute for Tumor Research Chungbuk National University Cheongju Korea

**Keywords:** Bmp pathway, Bmp6, liver iron metabolism, Runx3

## Abstract

**Objectives:**

Runx3, a member of the Runx family of transcription factors, has been studied as a tumour suppressor and key player of organ development. In a previous study, we reported differentiation failure and excessive angiogenesis in the liver of *Runx3* knock‐out (KO) mice. Here, we examined a function of the Runx3 in liver, especially in iron metabolism.

**Methods:**

We performed histological and immunohistological analyses of the *Runx3* KO mouse liver. RNA‐sequencing analyses were performed on primary hepatocytes isolated from *Runx3* conditional KO (cKO) mice. The effect of *Runx3* knock‐down (KD) was also investigated using siRNA‐mediated KD in functional human hepatocytes and human hepatocellular carcinoma cells.

**Result:**

We observed an iron‐overloaded liver with decreased expression of hepcidin in *Runx3* KO mice. Expression of BMP6, a regulator of hepcidin transcription, and activity of the BMP pathway were decreased in the liver tissue of *Runx3* KO mice. Transcriptome analysis on primary hepatocytes isolated from *Runx3* cKO mice also revealed that iron‐induced increase in *BMP6* was mediated by Runx3. Similar results were observed in *Runx3* knock‐down experiments using HepaRG cells and HepG2 cells. Finally, we showed that Runx3 enhanced the activity of the *BMP6* promoter by responding to iron stimuli in the hepatocytes.

**Conclusion:**

In conclusion, we suggest that Runx3 plays important roles in iron metabolism of the liver through regulation of BMP signalling.

## Graphical abstract

The increasing body iron stimulates the production of BMP6, which binds to a complex of type I and II BMP receptors on the plasma membrane of hepatocytes. This leads to the phosphorylation of SMAD1, 5 and 8 in the cytoplasm, which allows the binding of SMAD4. The entire complex is then translocated into the nucleus where it binds to BMP responsive elements in the hepcidin promoter, stimulating transcription. However, the regulation mechanism of BMP6 by iron stimuli has not yet been revealed. Here, we showed that *Runx3* KO or KD in mouse primary hepatocytes, human hepatocytes and human hepatocarcinoma decreased BMP6 expression and inhibited the BMP‐Smad pathway in human hepatocytes. Therefore, Runx3 regulates iron metabolism of the liver via modulation of BMP signaling.

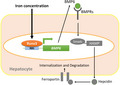

## 1 INTRODUCTION

Iron is crucially involved in many of the essential physiological processes of the human body, such as oxygen delivery by red blood cells, energy processes in muscles and enzymatic catalysis of various metabolic processes.^1^ Intestinal cells release absorbed iron from the diet into the plasma, and iron binds with transferrin, an iron carrier protein, in the plasma to be distributed to the target organ or cells. However, excessive iron release into the plasma can saturate the binding capacity of transferrin and result in non‐transferrin‐bound iron in the blood, which is a highly reactive form that can cause cellular and visceral damage.^2^ Therefore, tight regulation of plasma iron is required to avoid iron‐related toxicity in the body.

Hepcidin, a key regulator of iron transport, suppresses the release of iron from macrophages or intestinal cells into the plasma via binding to ferroportin, which induces internalization and degradation of the cellular iron exporter.^3^ Genetic deficiency of hepcidin causes excessive iron in blood, which is followed by the deposition of iron and consequent functional failure in the liver and other tissues.^4^, ^5^, ^6^, ^7^ The bone morphogenetic protein (BMP) signalling pathway is a major regulatory pathway of hepcidin expression in the liver.^8^, ^9^ In hepatocytes, the pathway is initiated by the binding of BMP6 with the BMP receptor (BMPR) complex and a membrane‐anchor co‐receptor hemojuvelin (HJV) at the cell surface.^8^ The binding elevates kinase activity of the BMPR complex and results in phosphorylation of Smad1, Smad5 and Smad8, the cytoplasmic effectors of the BMP pathway. Phosphorylated Smad1, 5 and 8 form heteromeric complexes with the common mediator Smad4, and they then translocate into the nucleus to induce the transcription of target genes.^10^ A deficiency of the BMP pathway‐related genes causes low hepcidin expression, excessive iron in the blood and iron‐overloaded organs in mice.^11^, ^12^, ^13^, ^14^, ^15^, ^16^

The activity of the BMP signalling pathway in the liver should be associated with the plasma iron concentration to maintain iron homeostasis. In the liver of mice fed high‐iron diet, transcriptional activation of *BMP6* has been observed.^13^ Recent studies suggested liver sinusoidal endothelial cells (LSECs) as main sources of hepatic BMP6 responding to the iron stimuli.^17^, ^18^ Hepatocytes, once considered to serve dual roles as iron‐sensor and autocrine sources of BMP6, revealed as passive producers of hepcidin regulated by paracrine BMP6 from non‐parenchymal cells.^19^ However, high expression of transferrin receptor 2 (TfR2) and its unveiled function in hepatocytes^18^ implies possible mechanism of direct sensing of iron by the hepcidin producer.

Here, we demonstrated that Runx3 is an upstream regulator of *BMP6* in the liver. Prussian blue staining revealed an iron‐overloaded liver at postnatal day 1 (PN1) in *Runx3* knock‐out (KO) mice. Hepcidin was decreased in the liver of *Runx3* KO mice. Interestingly, a similar iron‐overloaded liver was reported in *Bmp6* KO mice.^11^ To reveal the possible engagement of BMP signalling with Runx3 deficiency‐induced iron overload in the liver, we detected BMP6 expression in the liver tissue. The results showed a decrease in BMP6 and BMP signalling in the liver of *Runx3* KO mice. A systematic approach using RNA sequencing of primary hepatocytes isolated from *Runx3* conditional KO (cKO) mice revealed that *BMP6* was specifically induced by iron stimuli, and *Runx3* KO using Cre recombinase‐expressing adenovirus aborted the iron‐induced *BMP6* expression in the hepatocytes. Down‐regulation of these genes was also observed in *Runx3* knock‐down (KD) in both HepaRG cells, which are functional human hepatocytes,^20^ and HepG2 cells, which are human hepatocellular carcinoma cells.^21^ The *Runx3* KD abolished iron‐induced *BMP6* transcription and the resultant activation of BMP signalling in both cells. Furthermore, we found that Runx3 activated the promoter of *BMP6* to trigger the BMP signalling‐mediated hepcidin regulation by iron stimulation. Taken together, Runx3 plays important roles in the iron metabolism of the liver through regulation of BMP signalling.

## 2 METHODS

### 2.1 Runx3 KO and cKO mice

*Runx3* knock‐out (*Runx3* ^−/−^ FVB) and Runx3 cKO (*Runx3^flox^*/*^flox^* C57BL/6) mice were generated and maintained as described previously.^22^, ^23^ The animals were maintained in pathogen‐free conditions and monitored daily. All experiments were performed according to the guidelines of the Yonsei University College of Dentistry, Intramural Animal Use and Care Committee.

### 2.2 Histology and immunohistochemistry

Samples were fixed in 4% paraformaldehyde in phosphate‐buffered saline (PBS) and then embedded in paraffin using standard procedures. Serial paraffin sections (4‐μm thickness) were prepared, and individual slides were stained with haematoxylin and eosin. Antigen retrieval was achieved by citrate buffer, pH 6.0. After antigen retrieval, immunohistochemical analyses were performed using following primary antibodies: Hepcidin (ab‐75883; AbCam, Cambridge, UK), L‐Ferritin (ab‐69090; AbCam), Ferroportin‐1 (sc‐49668; Santa Cruz Biotechnology, Inc., Santa Cruz, CA), BMP‐6 (bs‐10090R; Bioss Antibodies, Bioss Antibodies, Woburn, MA) and Smad‐4 (sc‐7966; Santa Cruz Biotechnology, Inc.). Immunostainings were performed using the DakoCytomation Envision System (DAKO, Glostrup, Denmark) according to the manufacturer's instructions. Alexa Fluor‐conjugated secondary antibodies (Invitrogen, Carlsbad, CA) were used for immunofluorescent staining. The stained sections were examined with a stereomicroscope (MD5500D; Leica Microsystems, Wetzlar, Germany) and a confocal microscope (LSM700; Carl Zeiss, Jena, Germany).

### 2.3 Western blotting analyses

Liver tissue and hepaRG cells underwent lysis by sonication (Next Advance Inc., Averill Park, NY) in radio‐immunoprecipitation assay (RIPA) buffer (50 nM Tris pH 7.5, 150 mM NaCl, 1 mM EDTA, 1% Triton X‐100). Anti‐L‐Ferritin or anti‐α‐Tubulin (T6199; Sigma‐Aldrich, St. Louis, MO) antibody was used. Horseradish peroxidase‐conjugated secondary antibodies (Santa Cruz Biotechnology, Inc.) were used, and the protein bands were visualized by enhanced chemiluminescence (Amersham Biosciences, Piscataway, NJ).

### 2.4 Primary hepatocyte isolation and RNA preparation

Primary hepatocytes of *Runx3* cKO mice were isolated as described previously.^24^ The isolated hepatocytes were infected with Cre recombinase‐expressing adenovirus (Ad‐Cre‐GFP, #1700; Vector Biolabs, Philadelphia, PA) to induce knock‐out of *Runx3*. After 24 h of the virus treatment, 60 mM of holo‐transferrin (hTF, #616424, Millipore Corp., Bed ford, MA) was treated to the hepatocytes for 24 h. Total RNAs were isolated using TRIzol reagent as a manufacturer's instruction (#10296010; Invitrogen). RNA concentrations were quantified using a NanoDrop spectrophotometer (NanoDrop, Wilmington, DE), and the 260/280 nm ratio was confirmed to be between 1.7 and 2.0. The integrity of the total RNA samples was evaluated using the Agilent 2100 (Agilent Technologies, Inc., Santa Clara, CA) and Tecan F2000 (Tecan Group Ltd., Männedorf, Switzerland) devices, and only samples with an RNA integrity number (RIN) >7.0 and high‐quality RNA (28S/18S > 1) were used for the subsequent experiments.

### 2.5 RNA‐sequencing and data analysis

Reverse transcription was performed, and cDNA was synthesized using 5′ adaptor forward and 3′ adaptor reverse primers. Libraries for Illumina sequencing were constructed from cDNA as described.^25^ High‐throughput RNA sequencing was performed by Theragen Bio Institute (Suwon, Korea) on an Illumina HiSeq 2000 high‐throughput sequencer (Illumina, Inc. San Diego, CA) according to the manufacturer's specifications. RNA‐sequencing data were analysed according to the method described. Briefly, reads were mapped to the *Mus musculus* reference genome obtained from the University of California, Santa Cruz (UCSC) database using TopHat and Bowtie from Illumina iGenomes. Gene expression values were measured for each gene from the Ensembl database by fragments per kilobase of exon per million mapped reads (FPKM) calculated using Cufflinks.^26^ Differentially expressed genes were considered in a given library when the *p*‐value was less than 0.05 and a greater‐than‐or‐equal to twofold change in expression across libraries was observed and used to identify the genes differentially expressed between two samples. Clustered heat maps and volcano plots were drawn using a statistical computing software, R (https://www.R‐project.org/).

### 2.6 Cell culture, transfection and analysis

HepG2 cells were cultured in Dulbecco's Modified Eagle's Medium (DMEM; Invitrogen) supplemented with 10% foetal bovine serum (FBS; Invitrogen) and 1% penicillin/streptomycin (Invitrogen). Cells were transfected with Runx3 siRNA (Santa Cruz Biotechnology, sc‐37679) or a luciferase reporter plasmid containing BMP6 promoter (S710500; Switchgear Genomics, Menlo Park, CA) using Lipofecatmine (Invitrogen) as a manufacturer's instruction. For real‐time PCR, total RNA of cells were extracted using TRIzol reagent. The extracts were reverse‐transcribed using Maxime RT PreMix (#25081; iNtRON, Daejon, Korea). The products were subjected to real‐time PCR analyses with primer sets designed using Primer Express software (Applied Biosystems, Foster City, CA) and StepOnePlus Real‐Time PCR System (Applied Biosystems). For reporter assay, cells were lysed and reacted using Luciferase Assay System (Promega, Madison, WI) and the luciferase activities were measured using Centro XS3 Microplate Luminometer LB 960 (Berthold Technologies, Oak Ridge, TN).

## 3 RESULTS

### 3.1 Iron overload in Runx3 KO mouse liver hepatocytes at PN1

**FIGURE 1:**
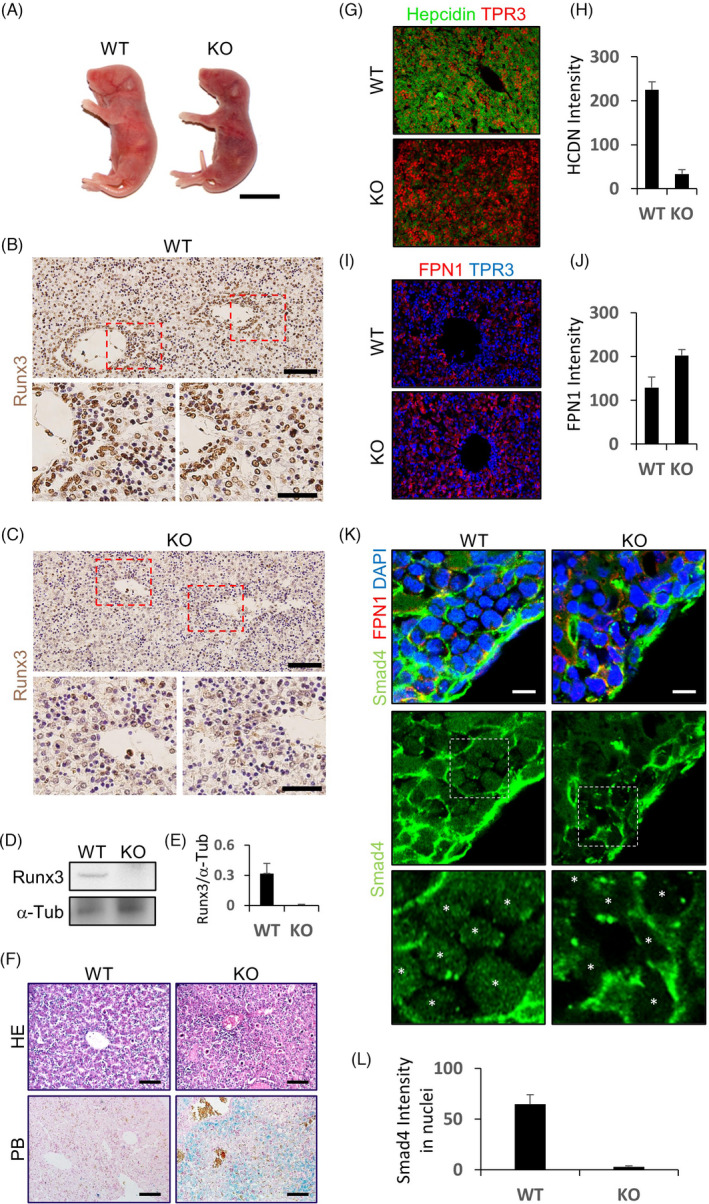
*Runx3* knock‐out mice showed a haemochromatosis‐like phenotype. (A) Wild‐type (WT) and *Runx3* knock‐out (KO) mice postnatal day 1 (PN1). Scale bar =1 cm. (B)–(L), The liver tissues of PN1 WT and KO mice isolated and subjected to histological staining, immunohistological staining or immunoblot analyses. The expressions of Runx3 (B)–(C), hepcidin (G), ferroportin1 (FPN1, I and K) or Smad4 (K) in the tissue sections were visualized by 3,3'‐diaminobenzidine (DAB, B and C) or fluorescence (G, I, and K) staining. Haematoxylin and eosin (HE, F, upper panels) and Prussian blue (PB, F, lower panels) staining of the tissue sections. Nuclei were counterstained using haematoxylin (B, C and F), TO‐PRO‐3 (TPR3, G and I) or 4’,6‐diamidino‐2‐phenylindole (DAPI, K). Scale bar =100 µm (B and C upper panels), 50 µm (B and C lower panels), 25 µm (F) or 10 µm (K). Asterisks are indicated nuclei of the cells (K). Quantifications performed using 3 immunoblot or immunostaining results obtained from liver tissues from 3 individual WT and *Runx3* KO mice (E, H, J and L)

*Runx3* KO mice showed lethality soon after birth, as reported previously.^27^ At postnatal day 1, skin pigmentation was observed in the KO mice (Figure 1A), which revealed a possible abnormality in iron metabolism.^28^ Depletion of Runx3 in the liver tissue of *Runx3* KO mice was confirmed using immunohistochemistry and immunoblotting (Figure 1B–1E). Using Perls’ Prussian blue staining, substantial iron accumulation was visualized in liver parenchymal cells (hepatocytes) of *Runx3* KO mice at PN1 (Figure 1F). The accumulation of iron was significantly higher in the central lobule region than the peripheral vein region of the liver (Figure 1F). In addition, an increase in ferritin protein, indicating accumulation of iron in the cytosol, was observed in the hepatocytes of *Runx3* KO mice compared to those of WT mice (Figures S1A, S1B).

The iron accumulation in the centrolobular region of liver tissue is a typical pattern of iron overload caused by hepcidin deficiency.^29^ Therefore, we examined hepcidin expression in the liver tissue of *Runx3* KO mice. The expression of hepcidin decreased in the liver tissue of *Runx3* KO mice compared to that of WT mice (Figure 1G, 1H). On the contrary, ferroportin expression was increased in the KO mouse liver, indicating escape of the iron exporter from hepcidin‐mediated degradation (Figure 1I, 1J).

To investigate possible engagement of BMP signalling in the iron‐overloaded liver of *Runx3* KO mice, BMP signalling activation in the liver tissue. Nuclear Smad4, an indicator of BMP signalling activation, was decreased in the hepatocytes of *Runx3* KO mice compared with those of WT mice (Figure 1K, 1L).

### 3.2 Systemic regulation of BMP signalling‐ and iron metabolism‐related genes by Runx3 KO in primary hepatocytes

**FIGURE 2:**
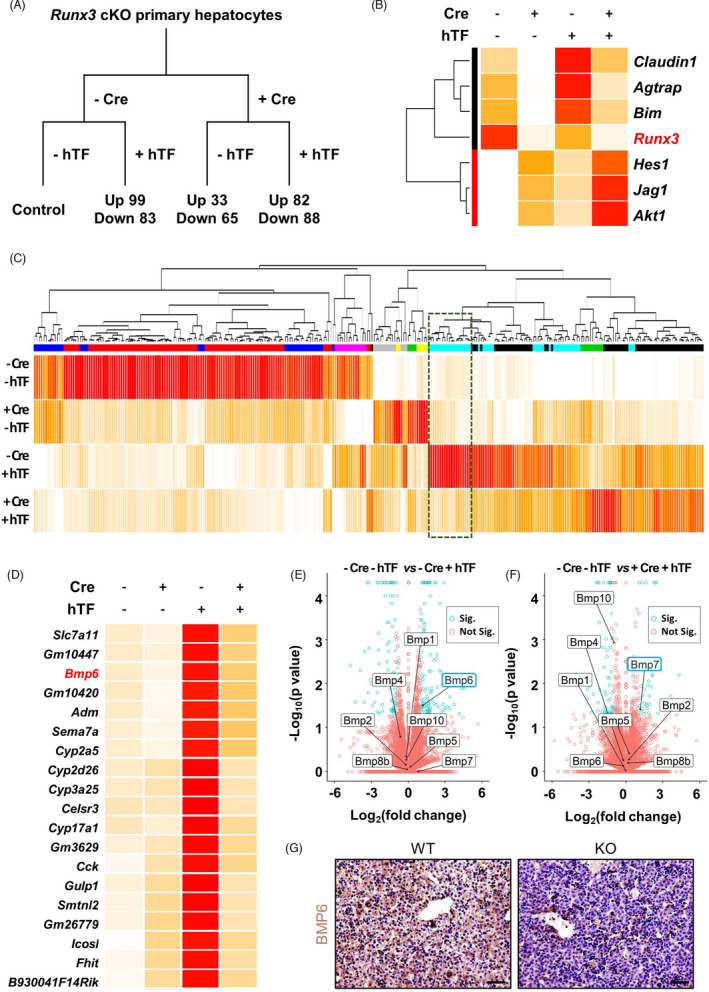
Induction of *Runx3* KO altered transcriptome of primary hepatocytes incubated with or without holo‐transferrin treatment. (A) A schematic diagram of RNA‐sequencing analysis. Holo‐transferrin (hTF) was treated (+hTF) or not treated (−hTF) on primary hepatocytes isolated from *Runx3* conditional knock‐out (cKO) mice infected (+Cre) or not infected (−Cre) with Cre recombinase‐expressing adenovirus. Differently expressed gene (DEG) analysis revealed the number of significantly upregulated (Up) or down‐regulated (Down) genes (fold change >2 and *p*‐value <0.05) in the 3 treated groups (−Cre +hTF, + Cre −hTF and +Cre + hTF) compared to the control group (−Cre–hTF). (B) A clustered heat map of the expression of *Runx3* and direct target genes of Runx3. (C) A clustered heat map of 294 genes significantly changing in at least one treated group. A dashed‐line box indicates a cluster of genes showing high sensitivity to iron stimuli. (D) A heat map of 19 genes showing high sensitivity to iron stimuli. (E) and (F) The results of DEG analyses displayed as volcano plots. The genes showed significant changes (Sig., fold change >2 and *p*‐value <0.05) or not significant changes (Not Sig., fold change <2 and *p*‐value >0.05), which were indicated as green and red points respectively. The *BMP* family genes are indicated by arrows and names. The blue line box indicates significantly changed genes of *BMP* family genes. E. The liver tissues of PN1 WT and KO mice isolated and subjected to immunohistological staining. The expressions of BMP6 in the tissue sections were visualized by DAB. Nuclei were counterstained using haematoxylin. Scale bar =50 µm

The lethality of *Runx3* KO mice set limitations on the systemic analysis of the Runx3 effect on iron metabolism. We introduced cKO mice of *Runx3* to avoid the limitations.^22^ Primary hepatocytes of *Runx3* cKO mice were isolated, and *Runx3* was knocked out by exogenous expression of Cre recombinase (Cre) using an adenoviral expression system (Figure 2A). The *Runx3* KO or control hepatocytes were incubated with or without hTF, a complex of iron and its carrier protein (Figure 2A). The alternation of transcriptomes by iron stimuli in the cells was monitored by RNA‐sequencing analysis (Figure 2B–2H).

The analysis result showed that Runx3 was knocked out in Cre overexpressed hepatocytes (Figure 2B). We examined expressions of the direct target genes of Runx3, which are known to be positively (*Claudin1*, *angiotensin II type 1 receptor*‐*associated protein* [*Agtrap*] and *Bim*)^30^, ^31^, ^32^ or negatively (*Hes1*, *Jagged1* [*Jag1*] and *Akt1*)^33^, ^34^, ^35^ regulated by Runx3 (Figure 2B). As expected, *Claudin1*, *Agtrap* and *Bim* were decreased and *Hes1*, *Jag1* and *Akt1* were increased by the *Runx3* KO (Figure 2B).

Differentially expressed gene (DEG) analysis revealed that 182 (up 99 and down 83), 98 (up 33 and down 65) and 170 (up 82 and down 88) genes were significantly regulated by Cre overexpression, hTF treatment or both respectively (Figure 2A and Tables S1 and Table S2). The expression patterns of 294 genes, which were significantly regulated in at least one treated group, were visualized as a heat map (Figure 2C). The clustered heat map showed a cluster of genes that were highly responsive to hTF treatment (Figure 2C, dashed‐line box). Cre‐mediated *Runx3* KO suppressed the hTF‐induced expression of the 19 genes in this cluster (Figure 2C, 2D). *BMP6* was identified as one of the 19 genes (Figure 2D). This cluster also included genes that encode for cytochrome P450s, which are the liver‐specific heme‐containing enzymes (*Cyp2a5*, *Cyp2d26*, *Cyp3a25* and *Cyp17a1*); a sodium‐independent cystine‐glutamate antiporter involving ferroptosis (*Slc7a11*)^36^; and proteins related to gastric secretion and iron absorption in the stomach, *Adrenomedullin* (*Adm*)^37^ and *Cholecystokinin* (*Cck*)^38^ (Figure 2D).

To investigate the specificity of *BMP6* regulation by hTF treatment and Cre expression, we displayed the DEG analysis results on volcano plots and indicated all identified *BMP*s on the plots (Figure 2E, 2F). Most of the *BMP* members were identified in both control and hTF‐treated hepatocytes; however, only *BMP6* was significantly increased by the iron stimuli (Figure 2E). The iron‐induced expression of *BMP6* was not observed in *Runx3* KO hepatocytes (Figure 2F). Interestingly, *BMP7*, a potent substitute for BMP6 in iron metabolism,^39^ was significantly increased by hTF treatment in the *Runx3* deficient hepatocytes; however, the expression of other *BMP*s was not significant (Figure 2F). *Id1* and *Hepcidin*, target genes of the BMP signalling pathway, were not significantly changed by hTF treatment or Cre overexpression (Figures S2A, S2B). The opposite expression pattern of *BMP7* compared with *BMP6* possibly compensates for the *BMP6* deficiency by Runx3 KO. The increase in BMP6 was confirmed by immunohistochemistry of BMP6 in liver tissue of WT and *Runx3* KO mice (Figure 2G).

### 3.3 Regulation of BMP signalling‐ and iron metabolism‐related genes by Runx3 KD in human hepatocytes and hepatocellular carcinoma cells

**FIGURE 3:**
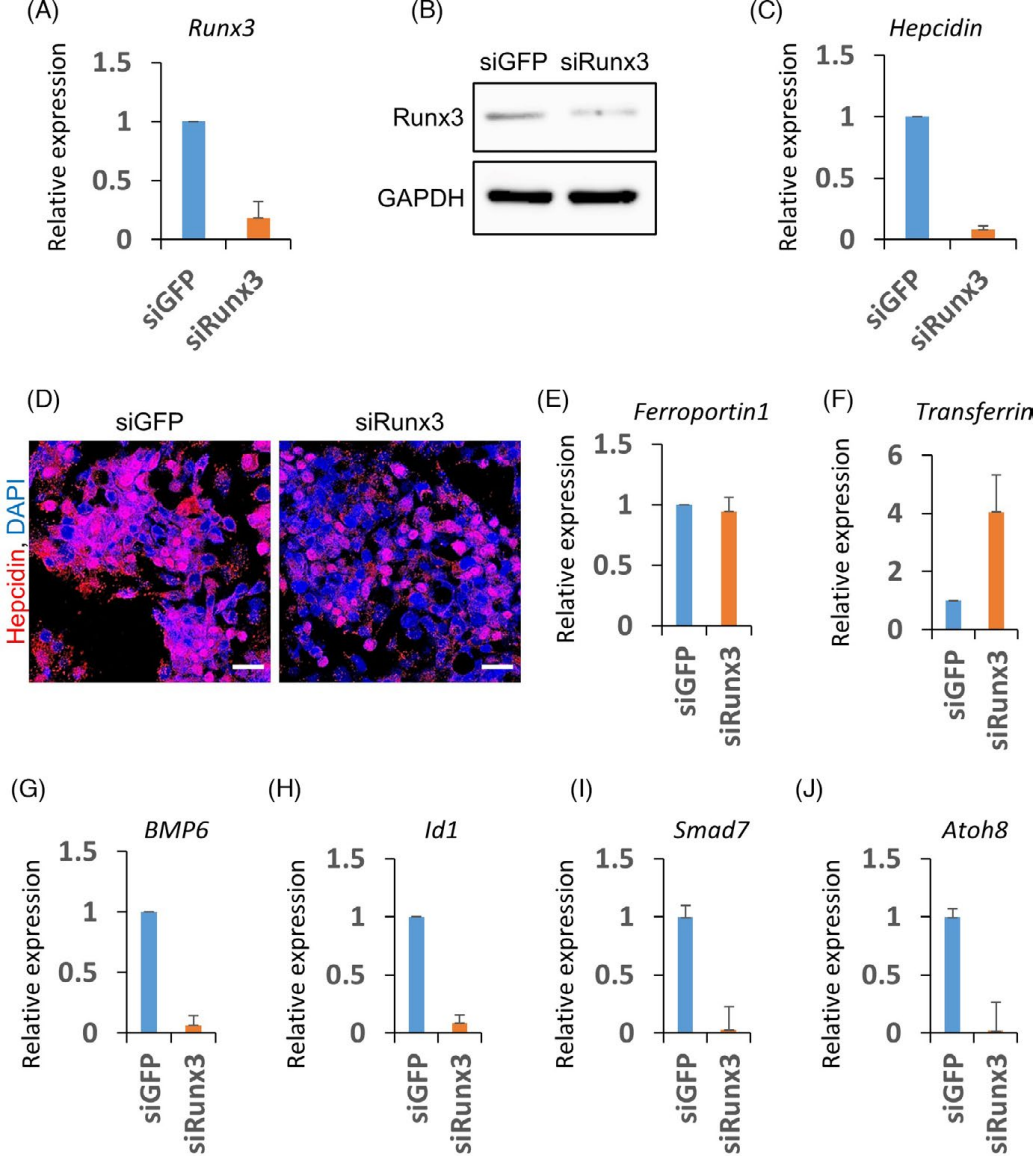
Runx3 knock‐down suppresses expression of BMP6‐ and iron metabolism‐related genes. (A)–(I), HepaRG (A‐C and E‐J) or HepG2 (D) cells were transfected with control (siGFP) or *Runx3* siRNA (siRunx3). The relative expressions of *Runx3*, hepcidin, ferroportin1, transferrin, *BMP6*, *Id1*, *Smad7* and *Atoh8* in the cells were measured by quantitative real‐time PCR (A, C and E‐J). Immunoblotting of siGFP or siRunx3‐transfected cells was performed using anti‐Runx3 and anti‐GAPDH antibodies (B). Immunocytochemistry using anti‐hepcidin antibodies visualized hepcidin expression in siGFP or siRunx3‐transfected cells (D, red). Nuclei were counterstained using DAPI (D, blue). Scale bar =20 µm

Here, we aimed to confirm the role of Runx3 on the expression of BMP signalling‐ and iron metabolism‐related genes using established hepatocyte cell lines. Firstly, we used HepaRG cells, which are functional human hepatocytes. Transfection of *Runx3* siRNA successfully decreased the mRNA level of *Runx3* in the hepatocytes (Figure 3A). The knock‐down effect also confirmed in protein level using immunoblotting (Figure 3B). Similar to the observation in the KO mice, the mRNA level of hepcidin decreased by the knock‐down (KD) of *Runx3* (Figure 3C). The *Runx3* KD effect also confirmed using HepG2 cells, which are human hepatocellular carcinoma cells (Figure 3D). Immunohistochemistry results revealed a decrease in hepcidin expression in the *Runx3* KD hepatocellular carcinoma cells (Figure 3D).

Hepcidin regulates ferroportin expression at the protein level, rather than at the transcription level.^3^ We found that *Runx3* KD cannot alter the mRNA level of ferroportin (Figure 3E). The mRNA level of transferrin, a liver‐originated iron carrier protein, increased by *Runx3* KD in the HepaRG cells (Figure 3F). To monitor the activity of the BMP pathway in *Runx3* KD hepatocytes, we detected the mRNA levels of the BMP ligand and BMP pathway target genes. The quantitative real‐time PCR results showed that mRNA levels of *BMP6* decreased by *Runx3* KD (Figure 3G). The mRNA levels of well‐known target genes of the pathway *Id1*, *Smad7* and *Atoh8* also decreased in the Runx3 KD hepatocytes (Figure 3H–3J).

### 3.4 Direct transcriptional regulation of BMP6 by Runx3

**FIGURE 4:**
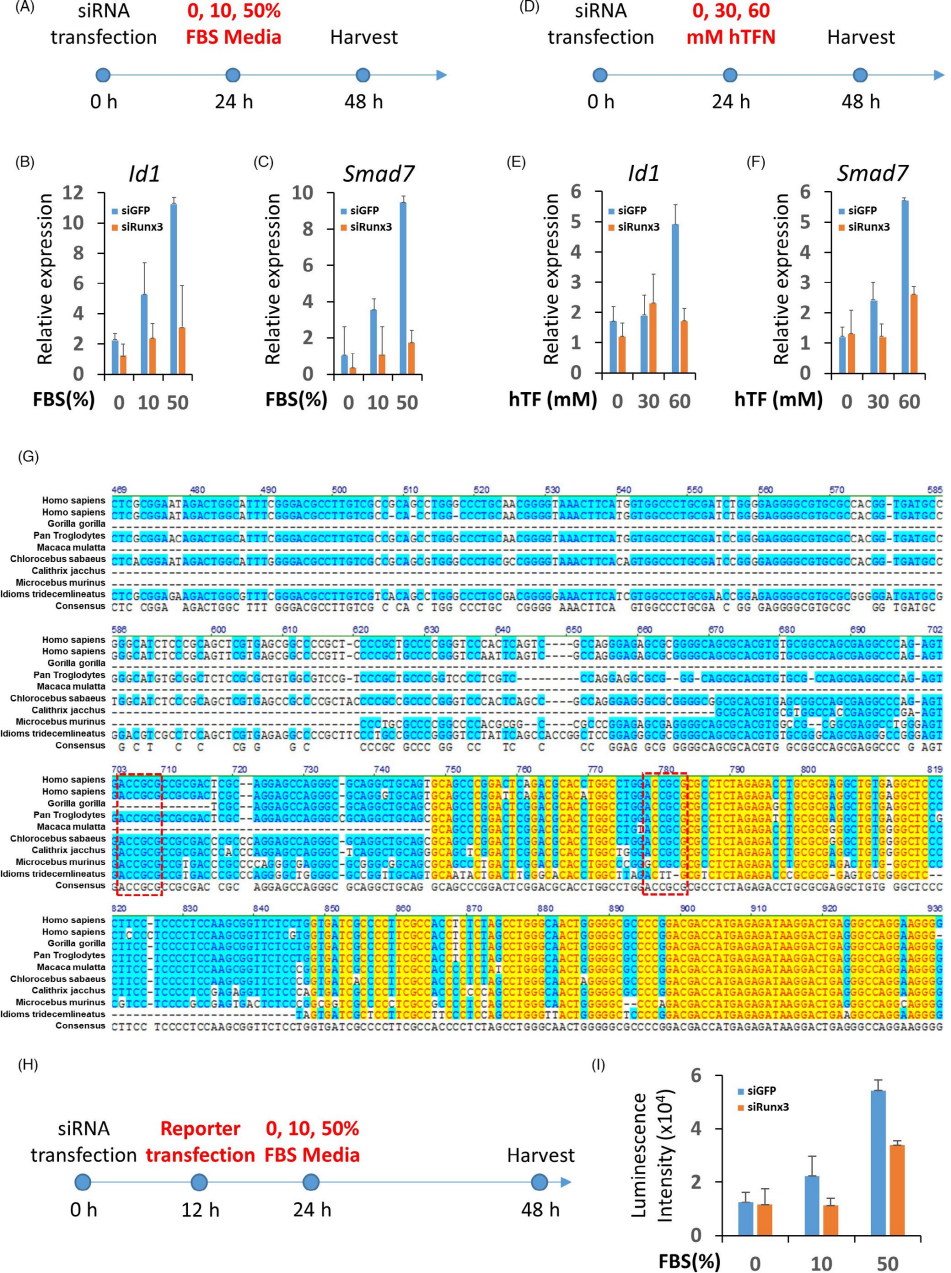
Runx3 mediates iron stimuli to direct regulation of *BMP6* expression. (A)–(F), Foetal bovine serum (FBS, A‐C) or hTF (D)–(F) of indicated doses was used to treat 24‐h starved HepG2 cells transfected with control siRNA or *Runx3* siRNA. The relative expressions of *Id1* and Smad7 were measured with quantitative real‐time PCR (B‐C and E‐F). G, An alignment result of BMP6 promoters of various species. Similar or identical amino acid sequences are indicated by blue or yellow blocks respectively. Putative Runx3 binding sites (RBSs) are indicated by a red dash‐lined box. H‐I, FBS of indicated doses were used to treat 24‐h starved HepG2 cells transfected with control siRNA or *Runx3* siRNA with a plasmid for the reporter assay of *BMP6* promoter. The activities of the BMP6 promoter were measured by luciferase activities (I). Control or *Runx3* siRNA‐treated groups are indicated by blue and red bars respectively (B‐C, E‐F and I)

BMP6 induction and activation of the BMP pathway by iron stimuli in hepatocytes were reported previously.^40^ To investigate whether Runx3 functions as a mediator between iron stimuli to BMP pathway activation, HepG2 cells transfected with control siRNA or *Runx3* siRNA were starved for 24 h to remove possible effect foetal bovine serum (FBS) in media and then treated with 0, 10 and 50% FBS for other 24 h (Figure 4A). The real‐time PCR results showed that the BMP pathway target genes, *Id1* and *Smad7*, were induced in a dose‐dependent manner with FBS treatment in control siRNA‐transfected cells (Figure 4B, 4C). However, activation of the BMP pathway was abolished in *Runx3* siRNA‐transfected cells (Figure 4B, 4C).

Holo‐transferrin is an iron‐bound form of transferrin, which is known to induce BMP6 expression.^41^ Similar to the experiment using serum, holo‐transferrin increases mRNA levels of *Id1* and *Smad7*; however, the effect was cancelled by *Runx3* KD (Figure 4D–4F). These results indicate that Runx3 functions as a mediator between iron stimuli and BMP pathway activation.

The Runt domain of the Runx3 has a specificity on a conserved sequence, 5′‐YGYGGT‐3′.^34^ Most of the target genes of Runx3 contain this sequence in their promoter region. We analysed an ~1 Kb upstream sequence from the BMP6 open reading frame to determine if the Runx3 binding sequence (RBS) existed, which was identified as one of the enriched motif in Runx3‐bound promoter of natural killer cells.^42^ The sequence analysis showed two putative RBSs in the BMP6 promoter region that were evolutionally well‐conserved (Figure 4G). We used a luciferase reporter plasmid containing the *BMP6* promoter to investigate the role of Runx3 on the transcriptional activity of the promoter (Figure 4H). Serum treatment showed a dose‐dependent increase of promoter activity in the reporter plasmid‐transfected HepG2 cells (Figure 4I). However, Runx3 KD suppressed activation of the BMP6 promoter induced by serum treatment (Figure 4I).

## 4 DISCUSSION

Hereditary haemochromatosis (HH) is a term used to describe a group of genetic disorders characterized by increased iron absorption.^43^ This absorption may lead to a progressive accumulation of iron in tissues and organs, resulting in impairment of organ structure and function, especially of the liver, pancreas, heart, pituitary gland and, likely, joints. The prevailing mechanism in most types of HH is deficiency of hepcidin, originally identified as an antimicrobial peptide^44^ and then shown to play a major role in iron homeostasis.^45^, ^46^ Hepcidin is synthesized mainly in hepatocytes and controls the plasma iron concentration by binding to ferroportin (also termed SLC40A1), the only known cellular iron exporter. After binding, ferroportin is degraded, reducing both intestinal absorption of iron from enterocytes and iron released from hepatocytes and macrophages. Increased plasma iron or cellular iron stores, as well as inflammation, generate a negative feedback loop that leads to a restriction of iron release into the plasma and blockade of dietary iron absorption through increased hepcidin production. In this study, *Runx3* KO mice showed a haemochromatosis‐like phenotype. Skin pigmentation and an iron‐overloaded liver were observed in the KO mice. Molecular biological analyses showed that BMP6 expression and activity of the BMP pathway were suppressed in the liver of *Runx3* KO mice.

**FIGURE 5:**
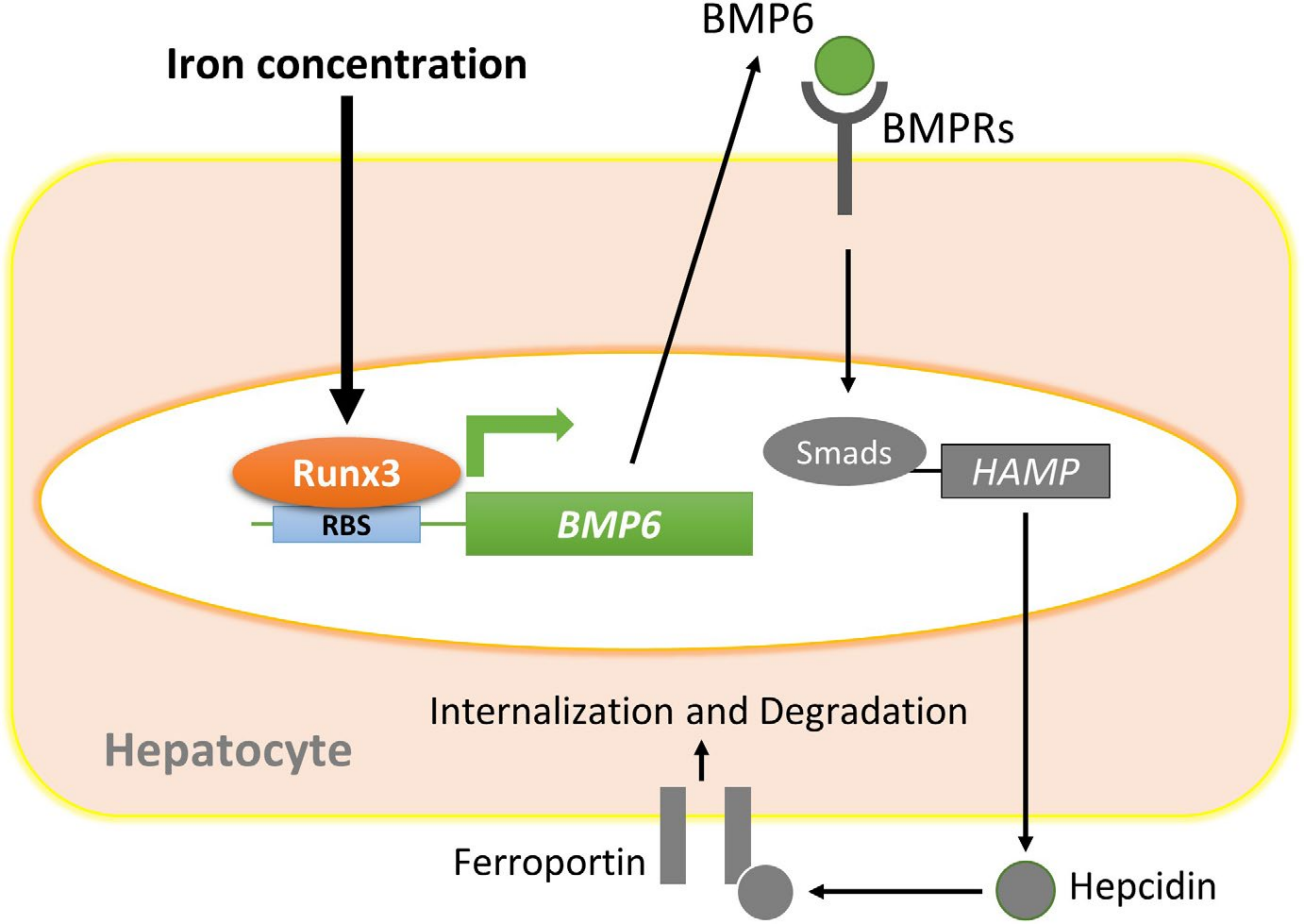
A schematic diagram of iron‐induced *BMP6* expression via Runx3 and the consequent regulation of hepcidin. The increasing body iron stimulates the production of BMP6, which binds to a complex of type I and II BMP receptors on the plasma membrane of hepatocytes.^11^, ^13^ This leads to the phosphorylation of SMAD1, 5 and 8 in the cytoplasm, which allows the binding of SMAD4. The entire complex is then translocated into the nucleus where it binds to BMP responsive elements in the hepcidin promoter, stimulating transcription. However, the regulation mechanism of BMP6 by iron stimuli has not yet been revealed. Here, we showed that *Runx3* KO or KD in mouse primary hepatocytes, human hepatocytes and human hepatocellular carcinoma decreased BMP6 expression and inhibited the BMP‐Smad pathway in human hepatocytes. Therefore, Runx3 regulates iron metabolism of the liver via modulation of BMP signalling

Studies have shown that, at least in rodent models, increasing body iron stimulates the production of BMP6, which binds to a complex of type I and II BMP receptors on the plasma membrane of hepatocytes (Figure 5).^11^, ^13^ This leads to the phosphorylation of SMAD1, 5 and 8 in the cytoplasm, which allows the binding of SMAD4. The entire complex is then translocated into the nucleus where it binds to BMP responsive elements in the hepcidin promoter, stimulating transcription. The glycosylphosphatidylinositol‐linked membrane protein HJV binds BMP6 and acts as a co‐receptor for the BMP receptor complex.^47^ HJV is essential for BMP6 signalling because the disruption of the protein, as occurs in the juvenile form of the iron loading disorder, haemochromatosis, leads to the complete loss of hepcidin production.^48^ However, the regulation mechanism of BMP6 by iron stimuli has not yet been revealed. Here, we showed that *Runx3* KO or KD in mouse primary hepatocytes, human hepatocytes and human hepatocellular carcinoma decreased BMP6 expression and inhibited the BMP‐Smad pathway in human hepatocytes. Therefore, Runx3 regulates iron metabolism of the liver via modulation of BMP signalling (Figure 5).

The upstream regulatory mechanism of Runx3 is remained to elucidated. Extra‐ or intra‐iron sensing proteins are possible candidates of a Runx3 modulator. The TfR2, a transferrin receptor, is functionally unknown although highly expressed in hepatocytes.^18^ Increase or decrease in intracellular iron induces redox change, which is recognized by redox proteins. The iron regulatory proteins, IRP1 and IRP2, possibly modulate Runx3 in a similar way with ferritin.^49^ Defining upstream modulator of Runx3 would be a first goal of future study.

Global transcriptome analysis of the *Runx3* KO primary hepatocytes with or without hTF treatment showed that the *BMP6* induction by iron stimuli was a specific regulation and that *Runx3* KO abolished the regulation. However, regulation of the target genes of the BMP‐Smad pathway was not observed in the transcriptome analysis. Of note, *BMP7*, another member of BMP ligands, showed the opposite pattern of expression to *BMP6*. BMP7 is closely related in structure to *BMP6* and shares the receptor complex to activate the BMP‐Smad signalling pathway.^50^ Furthermore, a previous study showed that *BMP7* was upregulated in the liver tissue of *BMP6* null mice treated with iron‐dextran, and exogenous BMP7 injected into the null mice induced hepcidin expression and reduced an abnormally high concentration of plasma iron.^39^ Therefore, a compensatory effect by BMP7 is a possible explanation for the insensitivity of the BMP‐Smad pathway in the experiment using primary hepatocytes.

## 5 CONCLUSION

In conclusion, this work depicts Runx3 as a transcription factor of regulating hepcidin expression. Our findings highlight possible role of Runx3 in human iron metabolism disorders, such as haemochromatosis, hemosiderosis and atransferrinemia.

## CONFLICT OF INTEREST

The authors declare that they have no competing interests.

## AUTHOR CONTRIBUTIONS

Hyun‐Yi Kim, Jong‐Min Lee, Shujin Li and Seung‐Jun Lee. carried out in vivo and in vitro experiments; Hyun‐Yi Kim, Jong‐Min Lee and Han‐Sung Jung designed the study and performed data analyses; You‐Soub Lee and Suk‐Chul Bae provided Runx3 cKO mice; Hyun‐Yi Kim, Jong‐Min Lee and Han‐Sung Jung wrote the manuscript, and Suk‐Chul Bae and Han‐Sung Jung reviewed the manuscript. All authors approved the author list, had access to the study data, and reviewed and approved the final manuscript.

## Supporting information

Fig S1
Fig S2

Table S1

Table S2

## Data Availability

The raw data supporting the conclusions of this article will be made available by the authors.
