# Supplementary material for: Runx3 regulates iron metabolism via modulation of BMP signalling
Source: Cell Prolif. 2021 Oct 6;54(12):e13138. doi: 10.1111/cpr.13138 (PMC8666273; doi:10.1111/cpr.13138)
Supplement: Supplementary file 1 — Fig S1 Fig S2 [file CPR-54-e13138-s002.docx]

**Runx3 regulates iron metabolism via modulation of BMP signaling**

Hyun-Yi Kim^1,*^ | Jong-Min Lee^1,*^ | You-Soub Lee^2^ | Shujin Li^1^ | Seung-Jun Lee^1^ | Suk-Chul Bae^2^ | Han-Sung Jung^1^

**Supplementary Figures and Figure legends**

**
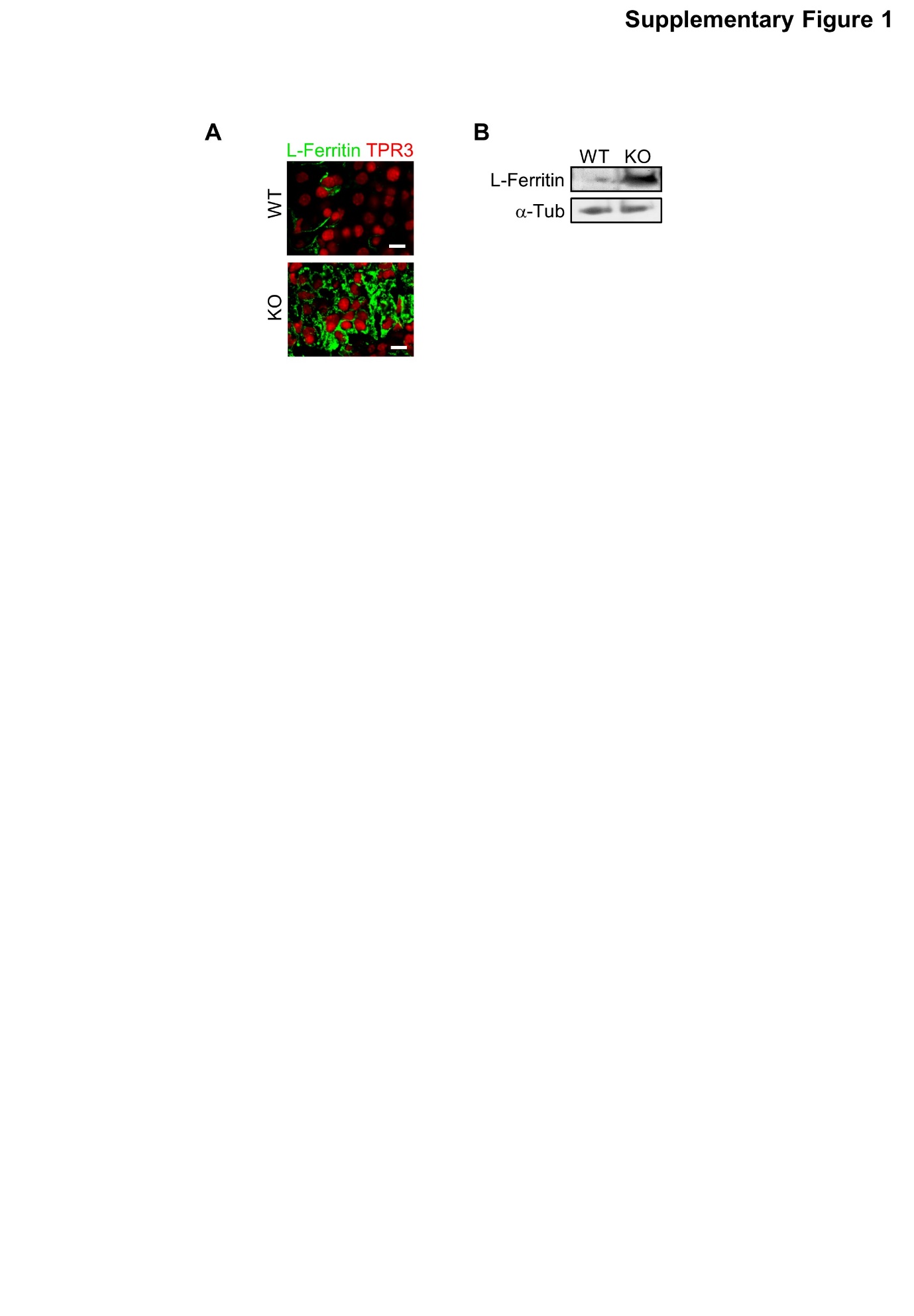
**

**Supplementary Figure 1** *Runx3* knock-out (KO) induces ferritin accumulation in cytosol of hepatocytes. A-B, The liver tissues of postnatal 1 wild-type (WT) and KO mice isolated and subjected to immunohistological staining (A) or immunoblot analysis (B) using anti-L-Ferritin and α-Tubulin (α-Tub) antibodies. Nuclei were counterstained using TO-PRO-3 (TPR3, A). Scale bar = 10 μm (A).


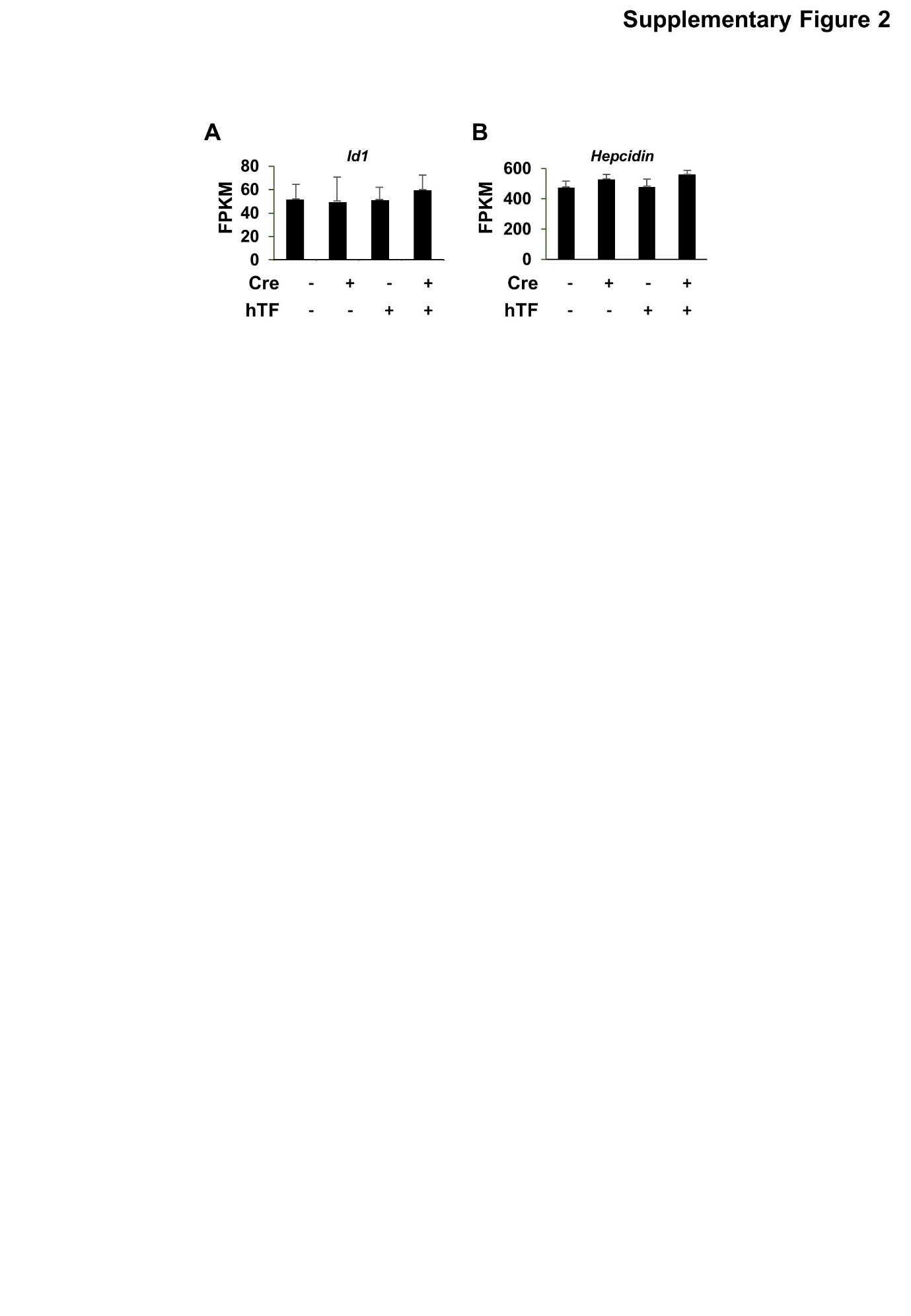


**Supplementary Figure 2** Effect of *Runx3* KO on transcription in primary hepatocytes incubated with or without holo-transferrin treatment. A-B, Holo-transferrin (hTF) was treated (+ hTF) or not treated (- hTF) on primary hepatocytes isolated from *Runx3* conditional knock-out (cKO) mice infected (+ Cre) or not infected (- Cre) with Cre recombinase expressing adenovirus. Graphs showing normalized quantities of *Id1* and hepcidin RNA (FPKM, fragments per kilobase million) in each group.
